# Direct Aggression and Generalized Anxiety in Adolescence: Heterogeneity in Development and Intra-Individual Change

**DOI:** 10.1007/s10964-015-0388-8

**Published:** 2015-12-09

**Authors:** Wim Meeus, Rens Van de Schoot, Skyler T. Hawk, William W. Hale, Susan Branje

**Affiliations:** Research Centre Adolescent Development, Utrecht University, PO Box 80.140, 3508 TC Utrecht, The Netherlands; Department of Developmental Psychology, Tilburg University, Tilburg, The Netherlands; Department of Methodology and Statistics, Utrecht University, Utrecht, The Netherlands; Optentia Research Program, Faculty of Humanities, North-West University, Mahikeng, South Africa; Chinese University of Hong Kong, Hong Kong, China

**Keywords:** Direct aggression, Generalized anxiety, Adolescence, Longitudinal research

## Abstract

Co-occurrence of aggression and anxiety might change during adolescence, or stay stable. We studied change and stability of four types of co-occurrence regarding direct aggression and anxiety in adolescence: an anxious and non-aggressive type, an aggressive and non-anxious type, a comorbid aggressive-anxious type and a no problems type. We applied a person-centered approach to assess increases and decreases of these types, and tested various models of intra-individual change of the types: the stability, acting out and failure models. We used data from a five-wave study of 923 early-to-middle and 390 middle-to-late adolescents (48.5 % male), thereby covering the ages of 12–20. We observed accelerated development in the older cohort: adolescents tended to grow faster out of the aggressive types in middle-to-late adolescence than in early-to-middle adolescence. We observed one other group-dependent pattern of heterogeneity in development, namely “gender differentiation”: gender differences in aggression and generalized anxiety became stronger over time. We found support for two perspectives on intra-individual change of the four types, namely the stability and the acting out perspective. The no problems—and to a lesser extent the anxious—type proved to be stable across time. Acting out was found in early-to-middle adolescents, males, and adolescents with poorer-quality friendships. In all three groups, there were substantial transitions from the anxious type to the aggressive type during 4 years (between 20 and 41 %). Remarkably, acting out was most prevalent in subgroups that, generally speaking, are more vulnerable for aggressive behavior, namely early-to-middle adolescents and males. We interpret acting out as the attempt of adolescents to switch from anxiety to instrumental aggression, in order to become more visible and obtain an autonomous position in the adolescent world. Acting out contributed to the explanation of accelerated development and gender differentiation. We also observed an increase of adolescents with no problems. These findings highlight that the co-occurrence of aggression and anxiety changes considerably during adolescence, but also that the anxious and no problems types are quite stable in this period.

## Introduction

Aggression and anxiety co-occur in childhood and adolescence. According to a recent review (Granic 2014), rates of anxiety disorders in conduct-disordered children and adolescents are at least 22 % in community samples and 60 % in clinic-referred samples. These co-occurrence rates suggest that it is useful to distinguish children and adolescents with strong co-occurrence (i.e., high levels of both aggression and anxiety) from those with weak co-occurrence (i.e., high levels of aggression and low levels of anxiety, or vice versa). Additionally, various studies have suggested that aggression and anxiety may affect each other over time (see for a review Bubier and Drabick 2009). Until now, however, no study has systematically looked into the development of co-occurring aggression and anxiety in adolescence. Hence, this study was specifically designed to do so. Our first aim was to provide a systematic account of the increase and decrease in the number of individuals with strong and weak co-occurrence of aggression and anxiety from early to late adolescence (ages 12–20). Secondly, we studied change and stability of aggression and anxiety within individuals in adolescence. We tested three models of stability and change in co-occurring aggression and anxiety: the stability, acting out and failure models. The stability model assumes that co-occurrence of aggression and anxiety does not change during adolescence, whereas the acting out model predicts that earlier anxiety will lead to later aggression in adolescence, and the failure model predicts that earlier aggression will lead to later anxiety. We used a person centered approach and five waves of longitudinal data.

### Development of Direct Aggression and Generalized Anxiety in Adolescence

#### Age-Effects

We will focus on direct aggression (defined as physical and verbal aggression towards others) and generalized anxiety disorder (defined as excessive, persistent, and uncontrollable worry). For a number of reasons, we decided to use specific conceptualizations of aggression and anxiety. For instance, many studies have shown that direct aggression differs from other forms of aggression. It is conceptually different from indirect and relational aggression (Cleverley et al. 2012; Crick et al. 1999), has higher rates of prevalence than indirect aggression (Cleverley et al. 2012) and has different associations with gender (Crick et al. 1999). Similarly, generalized anxiety disorder is conceptually distinct from other anxiety symptoms such as separation and social anxiety (Hale et al. 2005), becomes salient at different ages in adolescence (Weems 2008; Westenberg et al. 2001), and is linked to development in different domains (Nelemans et al. 2014). Using specific conceptualizations of aggression and anxiety will therefore give us a specific picture of the heterogeneity in their development.

Longitudinal studies have systematically shown that direct aggression decreases between the ages of 10 and 18 in adolescence (Bongers et al. 2004; Brame et al. 2001; Cleverley et al. 2012; Martino et al. 2008; Nagin and Tremblay 1999; Xie et al. 2011). Additionally, most of these studies identified various trajectories of aggressive behavior, ranging from consistently high levels of aggression to low levels of aggression. Aggression decreased in the majority of the trajectories, including in the high aggression ones. Thus, the normative pattern is a decrease of direct aggression in adolescence. This is most likely due to the fact that adolescents learn to settle conflicts without using direct aggression. For instance, their cognitive empathy increases (Van der Graaff et al. 2014) and they learn to use problem solving skills in conflicts (Van Doorn et al. 2011).

Fewer studies have addressed the development of generalized anxiety disorder in adolescence. Two studies followed subjects from early to late adolescence. Both Nelemans et al. (2014), who followed a subsample of 239 subjects in the younger cohort of the present study’s sample until ages 19.5, and Van Oord et al. (2009) found a curvilinear trend with an initial decrease of generalized anxiety and an increase from middle-adolescence. Only Nelemans et al. conducted trajectory analyses, and found a high and low anxiety trajectory. Both trajectories showed an increase in anxiety from middle adolescence onward, but slope factors were non-significant, probably due to a small sample size. The findings of both studies are consistent with theorizing that assumes that different anxiety symptoms become salient in different periods of childhood and adolescence (Weems 2008; Westenberg et al. 2001). For instance, according to this theorizing, separation anxiety becomes salient at the ages between 7 and 10 and decreases thereafter, whereas generalized anxiety becomes salient from middle adolescence onward. This is due to the fact that worry is at the heart of generalized anxiety, and that adolescents start worrying about the future from middle adolescence onward (Arnett 2000). In sum, we expected a decrease in direct aggression during adolescence and an increase in generalized anxiety disorder during middle-to-late adolescence.

#### Gender Differences

In general, all of the aforementioned studies reported higher levels of direct aggression in boys and higher levels of generalized anxiety in girls. Most of the studies found gender differences to be stable over time. Two studies tested explicitly whether gender differences in direct aggression increased during adolescence. Bongers et al. (2004) found that gender differences disappeared in adolescence, and Martino et al. (2008) reported stable gender differences. Nelemans et al. (2014) and Van Oord et al. (2009) report that generalized anxiety disorder grows faster in girls and no gender differences in growth, respectively. Taken together, these findings are consistent with the general observation that males show more externalizing problems such as direct aggression, and that females show more internalizing problems such as generalized anxiety disorder. We, therefore, expect to find more males in high direct aggression trajectories and more females in high generalized anxiety disorder trajectories. Less clear is whether gender differences in direct aggression and generalized anxiety decrease or grow in adolescence, respectively, or stay stable.

### Intra-Individual Change of Co-occurrence of Direct Aggression and Generalized Anxiety in Adolescence

In the literature, a number of theoretical models have been proposed to explain decreases in direct aggression and increases in generalized anxiety disorder during adolescence. These models assume intra-individual change of direct aggression and generalized anxiety: individuals grow out of using direct aggression and grow into generalized anxiety disorder during adolescence. The models, however, have been formulated to explain changes in broad categories of internalizing and externalizing problems (Angold and Costello 1993; Caron and Rutter 1991). Since direct aggression and generalized anxiety disorder are core to these broad categories (Krueger 1999), we believe that three of these models might be relevant for changes of direct aggression and generalized anxiety disorder into each other: the stability, failure and acting out models, respectively. A common assumption of these models is that internalizing and externalizing problems are caused by internal or external modulations of basic feelings of threat and fear (Krueger 1999). Theoretically, these modulations have been described as two types of evolutionarily selected reactions of individuals when confronted with threat, “fight” or “flight” (Nigg 2006). The three models, however, differ in their predictions regarding the development of internalizing and externalizing problems. The stability perspective posits that individuals maintain a stable style across time to cope with threat or fear. These styles constitute relatively stable individual traits: some individuals tend to fight and use externalizing reactions such as aggression, whereas others tend to use flight and react with internalizing problems such as anxiety. The *acting out perspective* holds that externalizing problems are basically behavioral manifestations of “masked” depression or anxiety (Carlson and Cantwell 1980). This masked anxiety might lead to aggression through loss of inhibitory control (Granic 2014). Therefore, the perspective predicts that internalizing problems will be expressed, or “acted out”, as externalizing problems over time. Hence, with respect to this study, this perspective would expect that earlier anxiety would lead to later aggression. Finally, and in opposition to the acting out perspective, the *failure perspective* predicts that earlier externalizing problems will lead to failure experiences, such as peer rejection or academic failure, and through them to internalizing problems. In light of this study, the failure perspective would predict that earlier aggression leads to later anxiety.

A limited number of person-centered studies have tested the three theoretical models for the development of co-occurring conduct disorder/behavioral problems and anxiety. Half of the six studies we identified used samples of clinically referred adolescents (Burke et al. 2005; Lahey et al. 2002; Last et al. 1996), and the other half used samples from the general population (Bittner et al. 2007; Ialongo et al. 1994; Roza et al. 2003). One study found support for the stability perspective (Lahey et al. 2002). Support existed for the failure perspective in some studies (Burke et al. 2005; Ialongo et al. 1994; Lahey et al. 2002; Roza et al. 2003). Finally, support was mixed regarding the acting out perspective, with some studies finding supporting evidence (Bittner et al. 2007; Last et al. 1996), and others finding no support (Burke et al. 2005). All studies used regression models to estimate effects, but none reported percentages of stability and change of individuals.

In sum, we might conclude that there is some support for the stability, failure and acting out perspectives. It is, therefore, difficult to draw final conclusions with regard to intra-individual stability and change of co-occurring aggression and anxiety over time in adolescence. Also, a final conclusion might not be reachable since most studies lacked the design to test the various theoretical models against each other. Therefore, the present article will compare these various perspectives in a single design, and will test them for direct aggression and generalized anxiety disorder. We will adopt a person-centered design, since we aim to study within-individual configurations of direct aggression and generalized anxiety disorder, as well as assess stability and change of these configurations within concrete individuals (Allport 1937, p. 48). We will, therefore, use a two-step approach. First, we will identify types of co-occurring (within-individual configurations) direct aggression and generalized anxiety disorder (aggression/anxiety types). Based upon our literature review we expect to find at least three aggression/anxiety types over time: a co-occurrence type high on both direct aggression and anxiety (from now on comorbid aggressive), and two weak-co-occurrence types, one high on direct aggression (aggressive), and one high on anxiety (anxious) only. Second, we will test the various theoretical perspectives by studying intra-individual stability and change in the types across time. Our approach allows us to determine the percentages of individuals that remain stably classified in the various aggression/anxiety types, the percentage of those who show failure (that is change from direct aggression to generalized anxiety), and those who show acting out (that is change from generalized anxiety to direct aggression). A variable-centered approach does not allow for this.

### The Role of Friendships

Notably, Patterson and colleagues (see for instance Capaldi 1992; Patterson and Stoolmiller 1992) have proposed that poor friendships are the key mechanism of failure. Externalizing problems of adolescents make them unattractive as friends, leading to poor friendships or peer rejection, which in turn leads to adolescent internalizing problems such as anxiety and depression. Support for this cascade model is mixed. Panak and Garber (1992) and Kiesner (2002) did not find evidence for the mediating role of friendships, whereas Van Lier and Koot (2010) and Pedersen et al. (2007) claimed to find such support. Therefore, we will study Patterson and colleagues’ assumption that failure is typical for adolescents with poorer-quality friendships. Since we aim to assess the role of personal relationships, we will focus on the relationship with best friend. From now on discussion of friendships refers to best friendships.

## The Present Study

The goal of our research was to study heterogeneity in the development of direct aggression and generalized anxiety disorder during adolescence. We addressed two sets of research questions. First, we studied development: is the prevalence of aggression/anxiety types stable, or do they increase or decrease during adolescence? Secondly, we tested patterns of change in types of direct aggression and generalized anxiety within individuals. If individuals change types, what do these changes look like? To meet these goals, we tested two hypotheses (Hs) and explored a number research questions when it was not possible to formulate hypotheses.

First, we addressed age, gender, and friendship differences in development of direct aggression and generalized anxiety disorder. Our first hypothesis (H1) originates from the review of research into normative development of direct aggression and generalized anxiety disorder in adolescence, and predicts a regular decrease of the aggressive type over time in adolescence, along with an increase of the anxious type in middle-to-late adolescence. We tested H1 for two cohorts: early-to-middle and middle-to-late adolescents. We explored increases and decreases of the strong co-occurrence type, the comorbid aggressive type. The second hypothesis (H2) addressed gender differences. In general, the studies of our literature review reported higher levels of direct aggression (Bongers et al. 2004; Brame et al. 2001) in boys and higher levels of generalized anxiety in girls Nelemans et al. 2014; Van Oord et al. 2009). Most of the studies found gender differences to be stable over time. However, Nelemans et al. (2014) reported that generalized anxiety disorder grows faster in girls. Taken together, these findings are consistent with the general observation that males show more externalizing problems such as direct aggression, and that females show more internalizing problems such as generalized anxiety disorder. We, therefore, expect to find more males in the aggressive type and more females in the anxious type. We explored whether gender differences in the prevalence of aggression/anxiety types are stable or increase in adolescence.

Secondly, we tested three models of intra-individual change in aggression/anxiety types: the stability, failure, and acting out models. To do so, we studied transitions between aggression/anxiety types. The stability model would predict individuals to stay stable in the various aggression/anxiety types over time. The failure model would predict that the earlier aggressive type develops into a later anxious type, and the acting out model that the earlier anxious type develops in a later aggressive type. We tested the research questions for early-to-middle and middle-to-late adolescents, for males and females, and adolescents with poorer- versus higher-quality friendships. The comparison of friendship groups allowed us to study Patterson and colleagues’ assumption that failure is typical for adolescents with poorer-quality friendships.

## Method

### Participants

Data for this study were collected as part of an ongoing Dutch research project on COnflict And Management Of RElationships (CONAMORE), with a 1-year interval between each of the five available waves. The longitudinal sample consisted of 1313 participants, divided into an early-to-middle adolescent cohort (*n* = 923; 70.3 %) with an average age of 12.4 years (*SD* = .59) at baseline, and a middle-to-late adolescent cohort (*n* = 390; 29.7 %) with an average age of 16.7 years (*SD* = .80) at baseline. Because both age groups were assessed during five measurement waves, a total age range from 12 to 20 years was available. The early-to-middle adolescent cohort consisted of 468 boys (50.7 %) and 455 girls (49.3 %), and the middle-to-late adolescent cohort consisted of 169 boys (43.3 %) and 221 girls (56.7 %). A more detailed description of the sample can be found in Meeus et al. 2011.

Sample attrition was 1.2 % across waves. In waves 1, 2, 3, 4, and 5, the number of participants was 1313, 1313, 1293, 1292 and 1275, respectively. We were able to keep attrition low by using a group of interviewers who collected data at home when the adolescents were not at school at the time of the annual measurement. Missing values of the study measures were estimated in SPSS, using the expectation maximization (EM) procedure. Little’s Missing Completely At Random (MCAR) test produced a normed χ^2^ (χ^2^/df) of 1.14, *p* > .05, which, according to Bollen (1989), indicates that the data were likely missing at random, and that it is safe to impute missing values.

### Procedure

Participants and their parents received an invitation letter describing the research project and goals and inviting them to participate. More than 99 % of the families who were approached signed the informed consent form. During regular annual assessments, participating adolescents completed questionnaires at school or at home. Confidentiality of responses was guaranteed. Adolescents received €10 (approximately US $13) for each wave in which they provided data.

### Measures

#### Generalized Anxiety Disorder

The 9-item Generalized Anxiety Disorder symptoms scale (from now on GAD) of the original 38-item SCARED (Screen for Child Anxiety Related Emotional Disorders) was employed in this study. This scale has been regularly used in developmental psychopathology studies of adolescents from the general population (Hale et al. 2005). Participants rated each symptom dimension item on a 3-point scale: 0 (almost never), 1 (sometimes), and 2 (often). A sample item is, “I worry about what is going to happen in the future”. Psychometric properties and concurrent validity of the generalized anxiety scale has been shown to be good (Nelemans et al. 2014). In the present study, internal consistency coefficients (Cronbach’s alphas) of the GAD scale ranged from .82 to .86 across waves.

#### Direct Aggression

The 5-item direct aggression scale of the Direct–Indirect Aggression Scale (DIAS) (Björkqvist et al. 1992) was used in this study. The questionnaire asks whether the adolescent would use physical and verbal aggression against somebody when the adolescent was angry with that person. The questions were scored on a scale from 1 (‘‘never’’) to 4 (‘‘always’’). Two sample questions are: ‘‘If I am mad or upset with someone …’’ ‘‘…I will call him (or her) names’’ and ‘‘…I will kick or hit him (or her)’’. Reliability and construct validity have been shown to be strong (Carroll and Schute 2005). Concurrent validity of the direct aggression scale was demonstrated in the present sample by significant associations with an often used Dutch measure (Baerveldt et al. 2003) of delinquency (correlations ranging between .38 and .43 across waves) and with the B5 trait agreeableness (correlations ranging between −.11 and −. 24 across waves). Cronbach’s alphas ranged from .84 to .91 across waves.

#### Quality of Best Friendships

The Network of Relationships Inventory (NRI) (Furman and Buhrmester 1985) was used to measure adolescents’ perceptions of support from best friend, negative interaction with best friend, and power of best friend. The support, negative interaction, and power scales consisted of 12, 6 and 6 items, respectively. The participants indicated their answers on a five-point Likert scale, ranging from 1 (*little or not at all)* to 5 (*more is not possible*). Examples of items are “Does your best friend like or approve of the things you do?” (support), “Do you and your best friend get on each other’s nerves?”(negative interaction), and “How often does your best friend tell you what to do?”(power). Internal consistencies were high, with alphas ranging across waves from .82 to .93 for support, negative interaction and power. Since we aimed to study the moderating role of best friendship quality in the development of aggression/anxiety, we used Latent class growth analysis (LCGA) to distinguish between higher- and poorer-quality friendships across the five waves. We estimated levels and linear and curvilinear changes of the three dimensions. We found a two-class solution to be superior to the one-class solution according to the bootstrapped VLMR-LMT test (*p* < .001). Entropy (E) of this solution was good, at .91. To avoid a large number of transitions with very low or zero cell frequencies in the latent transition (LTA) models, we decided to continue with the two-class solution and not to test for additional three or more class models. A majority of the 1045 respondents (79.4 %) was classified in the higher-quality friendship class, and 268 (20.6 %) in the poorer-quality friendship class. The higher-quality friendship class showed greater levels of support, lower levels of negative interaction, and lower levels of power, as compared to the poorer-friendship quality class.

### Analytic Strategy

To address our research questions, we used two applications of the general latent class model: latent class analysis (LCA) and latent transition analysis (LTA). LCA is a group-based, person-centered analytic strategy that is a confirmatory version of cluster analysis. LTA represents a longitudinal extension of LCA. LTA calculates increases and decreases in class size across time, as well as patterns of stability and change over time in the form of movement or transitions between classes (in this case, aggression/anxiety types). LTA offers two types of structural parameters: (a) varying numbers of participants in a class across waves, indicating increase or decrease in class size over time, and (b) transitions of individuals between classes that explain these changes in class size. Note that the transitions indicate patterns of intra-individual change in aggression/anxiety types. LTA is therefore appropriate for evaluating stability or change of the aggression/anxiety types over time, and the aggression/anxiety types transitions that explain stability and change over time.

We analyzed our data in three steps. First, we applied cross-sectional LCA to explore the number of classes (aggression/anxiety types) within each of the five waves. We found four classes in each of the waves. BIC of four-class solutions was at least 257.22 lower than that of one-, two-, and three-class solutions. Second, we select the best-fitting, five-wave LTA model in a number of successive steps. We found that a stationary, three-covariate LTA model provided the best fit to the data. BIC’s for the three-covariate model, and the no-covariate model were 10,541.02, and 10,677.21, respectively. This model suggests that adolescents regularly made transitions between aggression/anxiety types across waves, and with the same probability. Additionally, the regular pattern varied by age groups, gender, and best friendship groups. In sum, age groups, gender, and best friendship groups were moderators of these developmental processes. Entropy of the final model was very good (.87). Third, we applied Bayesian evaluations of the contingency tables generated by the final LTA model. LTA results can be converted into contingency tables that summarize the prevalence of classes (co-occurrence types) across waves. We use Bayesian Model Selection with (in)equality constraints between the parameters of interest (Klugkist et al. 2005) to evaluate these contingency tables. For a more detailed description of this method of analyzing contingency tables, readers are referred to Klugkist et al. 2010. In the Bayesian models, we used the transition probabilities of change in between waves 1 and 5. The 4-year probabilities were calculated using the contingency tables of waves 1–5, as generated by the final three-covariate LTA model. A detailed example of our three-step approach can be found in Meeus et al. (2011).

## Results

### Aggression and Anxiety Types Across Time in Adolescence

**Fig. 1:**
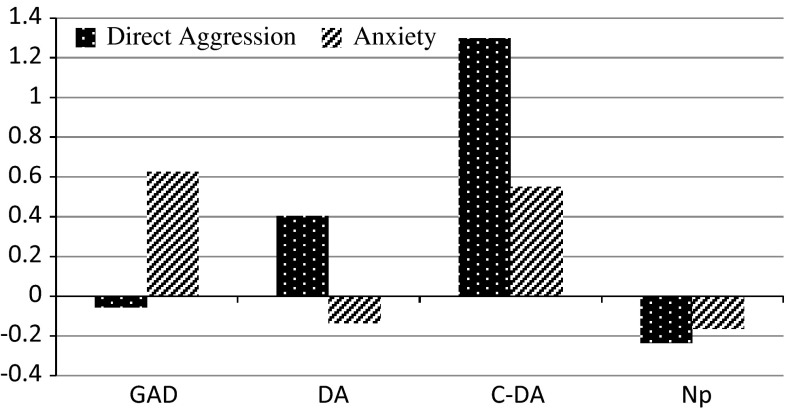
Profiles of the *GAD*, *DA*, *C-DA*, and *Np* classes on aggression and anxiety across waves. For reasons of presentation means were centered on grand mean of both means across waves (1.405)

Figure 1 displays the profiles of the classes found in the LCAs in each of the five waves. Class 1 is the anxious type, with a high score on anxiety symptoms and a low score on aggression (from now on labeled as GAD). Class 2 is the aggressive type, with a high score on direct aggression and a low score on anxiety (DA). Class 3 is the comorbid aggressive type, with very high levels of direct aggression combined with high levels of anxiety (C-DA). Class 4 is the no problems type, with low levels of both aggression and anxiety (Np). According to Dutch cut-off scores, anxiety levels in the GAD and C-DA types are in the upper part of the heightened anxiety category and below clinical levels (Muris et al. 2007). No standardized Dutch data are available for the aggression scores. Table 1, based on the final three-covariate LTA model, displays the cell sizes for each of the aggression/anxiety types for waves 1, 2, 3, 4, and 5 in early-to-middle and middle-to-late adolescents, males and females, and friendship groups.

**Table 1:** Size of classes for the whole sample, early-to-middle and middle-to-late adolescents, males and females, and adolescents with higher- and poorer-quality friendships

Wave	Aggression/anxiety types							
	Anxious		Aggressive		Comorbid aggressive		No problems	
	n	%	n	%	n	%	N	%
*Early*-*to*-*middle adolescence* (*n* = 923)								
1	107	11.6	255	27.6	81	8.8	480	52.0
2	105	11.4	278	30.1	78	8.5	462	50.1
3	120	13.0	286	31.0	69	7.5	448	48.5
4	133	14.4	265	28.7	57	6.2	468	50.7
5	125	13.5	263	28.5	54	5.9	481	52.1
*Middle*-*to*-*late adolescence* (*n* = 390)								
1	75	19.2	67	17.2	21	5.4	227	58.2
2	83	21.3	64	16.4	8	2.1	235	60.3
3	79	20.3	53	13.6	6	1.5	252	64.6
4	76	19.5	43	11.0	7	1.8	264	67.7
5	82	21.0	45	11.5	4	1.0	259	66.4
*Males* (*n* = 637)								
1	44	6.9	231	36.3	81	12.7	281	44.1
2	44	6.9	253	39.7	62	9.7	278	43.6
3	46	7.2	260	40.8	54	8.5	277	43.5
4	47	7.4	233	36.3	52	8.2	305	47.9
5	46	7.2	239	37.5	47	7.4	305	47.9
*Females* (*n* = 676)								
1	138	20.4	91	13.5	21	3.1	426	63.0
2	144	21.3	89	13.2	24	3.6	419	62.0
3	153	22.6	79	11.7	21	3.1	423	62.6
4	162	24.0	75	11.1	12	1.8	427	63.2
5	161	23.8	69	10.2	11	1.6	435	64.3
*Higher*-*quality friendships* (*n* = 1045)								
1	127	12.2	231	22.1	59	5.6	628	60.1
2	136	13.0	228	21.8	55	5.3	626	59.9
3	135	12.9	225	21.5	42	4.0	643	61.5
4	148	14.2	206	19.7	29	2.8	662	63.3
5	146	14.0	206	19.7	26	2.5	667	63.8
*Poorer*-*quality friendships* (*n* = 268)								
1	55	20.5	91	34.0	43	16.0	79	29.5
2	52	19.4	114	42.5	31	11.6	71	26.5
3	64	23.9	114	42.5	33	12.3	57	21.3
4	61	22.8	102	38.1	35	13.1	57	21.3
5	61	22.8	102	38.1	31	11.9	73	27.2

Findings based on the final stationary 1-year interval model with three covariates

### Development of Aggression and Anxiety in Adolescence

#### Age Group Differences in Increase or Decrease of Aggression/Anxiety Types Over Time (H1)

Visual inspection of panels 1 and 2 of Table 1 suggested differences between the early-to-middle adolescent and the middle-to-late adolescent cohorts. Decrease in DA was only apparent among the older cohort. Decrease in C-DA was stronger among the older cohort, as was increase in Np. Only the increase of GAD was similar in both cohorts. These findings did not support our original hypothesis (H1) that predicted a regular decrease of the aggressive type along with a regular increase of GAD in middle-to-late adolescence. We therefore tested a modified version of H1 that assumed differential increase and decrease in DA, C-DA, GAD, and Np types in both cohorts. We labeled the modified version of H1 as “accelerated development in the older cohort”, and used Bayesian Model Selection (Hoijtink 2012). We used panels 1 and 2 of Table 1 to test the hypothesis. Model 1 assumed no age group differences in increase or decrease of the four types across waves 1 and 5, whereas Model 2 assumed accelerated development in the older cohort. Specifically, this model assumed a greater decrease of both aggressive (DA) and comorbid aggressive (C-DA) types in middle-to-late-adolescence than in early-to-middle-adolescence and, consequently, a greater increase of the no problem (Np) type, along with a similar increase of the anxious (GAD) type in both cohorts. In Model 3, the unconstrained model, the increase and decrease in types across cohorts over time was allowed to vary freely; no constraints were specified between the four types in either cohort from wave 1 to 5, thereby assuming that every cell size was equally likely. First, Models 1 and 2 were compared with the unconstrained model (Model 3). The BFs for Models 1 and 2 implied that, after observing the data, these Models were approximately 1000 times less likely and 1156.44 times as likely, respectively, as the unconstrained model (Model 3). The second comparison revealed that Model 2 was 1156,440 times as likely as Model 1. Posterior model probabilities of Models 1, 2, and 3 were <.001, .99 and <.001, respectively. In sum, the “accelerated development in the older cohort” model (Model 2) supported the modified version of H1. Individuals tended to grow faster out of the aggressive and comorbid aggressive types in middle-to-late adolescence than in early-to-middle adolescence. On the other hand, chances to grow into the anxious type were similar in both cohorts. Model comparisons can be found in Table 2.

**Table 2:** Bayesian model selection: various sets of models on age group, gender and friendship differences in increase and decrease of aggression/anxiety types

Models	Model comparisons		
	BF		PMP
*Increase and decrease of aggression/anxiety types over time: age group differences*?			
M1. No age group difference in increase or decrease	<.001^a^	1	<.001
M2. Accelerated development in middle-to-late adolescence	1156.44	1,156,440	.99
M3. Unconstrained	1^b^		<.001
*Increase and decrease of aggression/anxiety types over time: gender differences*?			
M1. No gender difference in increase or decrease	.017	1	<.001
M2. Growth of gender differentiation	983.54	57,855.29	.99
M3. Unconstrained	1^b^		<.001

*BF* Bayes factor. *PMP* posterior model probability

^a^In the calculations of BFs the value was set at .001

^b^Models with BF = 1 are reference category

#### Gender Differences in Prevalence (H2) and Increase or Decrease of Aggression/Anxiety Types Over Time

Separate Chi square tests per wave showed that, in all waves, the prevalence of GAD was higher in females, and prevalence of DA was higher in males. These findings support H2. Additionally, prevalence of the C-DA type was higher in males (all tests *p* < .001, see also panels 3 and 4 of Table 1).

In addition, Table 1 shows subtle but systematic differences in change patterns between males and females. The increase of GAD and decrease of DA and C-DA was stronger among females than among males. We applied Bayesian Model Selection to test three alternative models of gender differences. Model 1 assumed no gender differences in increase or decrease of aggression/anxiety types from waves 1 to 5, whereas Model 2 assumed stronger increase in GAD and decrease in DA and C-DA among females. We labeled this model the “growth of gender differentiation” model, since it primarily indicated that existing gender differences in GAD, DA and C-DA become more pronounced as adolescents grow older. In Model 3, the unconstrained model, the distribution of aggression/anxiety types over time was allowed to vary freely across males and females. Model 2, the “growth of gender differentiation model”, was the best-fitting model. Thus, gender differences in GAD, DA, and C-DA became stronger over time. Model comparisons can be found in Table 2.

### Intra-Individual Change of Aggression/Anxiety Types Over Time: Stability, Failure and Acting Out

#### Stability

Transition probabilities of stability of the aggression/anxiety types across 4-year intervals, as found in the final stationary model, are presented in Table 3. The 4-year stabilities were calculated using the contingency tables of waves 1–5, as generated by the final three-covariate LTA model. The findings offered mixed support for the stability model. Stabilities of Np and, to a lesser extent, GAD were considerable, whereas those of DA and especially C-DA were relatively low. Stability of Np was higher than that of GAD, and stabilities of both Np and GAD were higher than those of DA and C-DA. Further, stabilities of Np and GAD were higher in middle-to-late than early-to-middle adolescents, females than males, and higher-quality than poorer-quality friendships (see Table 4). All Chi square tests were significant (at least *p* < .01).

**Table 3:** Stability of aggression/anxiety types during 4-year intervals across five waves in the whole sample

	GAD	DA	C-DA	Np
Four-year stability	.70	.49	.14	.80

Findings of the final stationary model with three covariates

**Table 4:** Transition probabilities of aggression/anxiety types during 4-year intervals across five waves for early-to-middle and middle-to-late adolescence, males and females, and higher- and poorer-quality friendships

DA/GAD type in year (n)	DA/GAD type in year (n + 4)				DA/GAD type in year (n + 4)			
	Early-to-middle adolescence				Middle-to-late adolescence			
	GAD	DA	C-DA	Np	GAD	DA	C-DA	Np
Anxious (GAD)	.58	.20	.03	.19	.88	.01	.00	.11
Aggressive (DA)	.05	.51	.10	.34	.03	.45	.06	.46
Comorbid aggressive (C-DA)	.10	.53	.17	.20	.19	.38	.00	.43
No problems (Np)	.08	.15	.02	.75	.04	.03	.00	.93

	Males				Females			
Anxious (GAD)	.41	.41	.02	.16	.80	.03	.01	.16
Aggressive (DA)	.04	.54	.12	.30	.08	.37	.03	.52
Comorbid aggressive (C-DA)	.10	.52	.13	.25	.19	.43	.14	.25
No problems (Np)	.04	.19	.03	.74	.09	.05	.01	.85

	Higher-quality friendships				Poorer-quality friendships			
Anxious (GAD)	.73	.06	.02	.19	.64	.25	.02	.09
Aggressive (DA)	.03	.48	.06	.43	.11	.51	.19	.19
Comorbid aggressive (C-DA)	.09	.52	.07	.32	.16	.47	.23	.14
No problems (Np)	.07	.09	.01	.83	.11	.27	.05	.57

Findings of the final stationary model with three covariates

^a^For a stationary model, all transitions probabilities are the same across waves. Transition probabilities sum up till 100 across rows. Transition probabilities can be interpreted as percentages. For instance the .58 of the GAD → GAD transition in early-to-middle adolescents indicate that 58 % of them stayed in GAD between wave 1 and wave 5 of the study

### Failure and Acting Out

#### Age Group Differences

The final three-covariate LTA model revealed age group differences in failure and acting out across 4-year intervals. Table 4, upper panel shows the results: failure was virtually absent and weaker than acting out in early-to-middle adolescence (DA → GAD_early-to-middle_ [.05] < GAD → DA_early-to-middle_ [.20]), whereas both failure and acting out were not apparent in middle-to-late adolescence (DA → GAD_middle-to-late_ = GAD → DA_middle-to-late_ [≤.05]). We applied Bayesian Model Selection to test three alternative models of age group differences of failure and acting out in waves 1 and 5. Model 1 assumed no cohort differences in failure and acting out. Model 2 assumed probabilities of failure to be lower than probabilities of acting out in the younger age group, as compared to no differences in probabilities in failure and acting out in the older age group. Model 3, the unconstrained model, did not specify any constraints of failure and acting out across age groups. Model comparisons showed that Model 2, the “acting out in early-to-middle adolescence model”, was by far the best-fitting model (see Table 5, upper panel). These findings suggested acting out to be stronger in the younger age group than in the older age group (see Table 4, upper panel: GAD → DA_early-to-middle_ [.20] > GAD → DA_middle-to-late_ [.01]). A follow-up set of Bayesian models confirmed this to be the case (Table 5, second panel).

**Table 5:** Bayesian model selection: various sets of models on age group, gender and friendship differences in transitions between aggression/anxiety types

Models	Model comparisons		
	BF		PMP
*Acting out versus failure different between age groups*?			
M1. No age group differences	<.02^a^	1	.001
M2. Acting out in early-to-middle adolescence	14.68	734	.935
M3. Unconstrained	1^b^		.064
*Acting out different between age groups*?			
M1. No age group differences	.007^a^	1	.002
M2. Acting out stronger in early-to-middle adolescence	2.03	290	.670
M3. Unconstrained	1^b^		.329
*Acting out versus failure different between genders*?			
M1. No gender differences	<.001^a^	1	<.01
M2. Acting out in males	3.73	3730	.78
M3. Unconstrained	1^b^		.21
*Acting out versus failure different between genders*?			
M1. No gender differences	<.001^a^	1	<.001
M2. Acting out stronger in males	2.02	2020	.66
M3. Unconstrained	1^b^		.33
*Acting out versus failure different between friendship groups*?			
M1. No differences between friendship groups	.90	1	.12
M2. Acting out in poorer friendships	5.44	6.04	.82
M3. Unconstrained	1^b^		.14
*Acting out different between friendship groups*?			
M1. No differences between friendship groups	.017	1	.006
M2. Acting out stronger in poorer quality friendships	2.01	118	.67
M3. Unconstrained	1^b^		.32

*BF* Bayes factor. *PMP* posterior model probability. Note that in some models the BF of model 2 is close to 2. This is due to the fact there is only one constraint imposed on the parameters in the model and as such the BF is limited to obtain a value of (approximately 2), see van de Schoot et al. (2011) for more details

^a^In the calculations of BFs the value was set at .001

^b^Models with BF = 1 are reference category

#### Gender Differences

The final three-covariate LTA model revealed gender differences in failure and acting out across 4-year intervals. Failure was found to be weaker than acting out in males (DA → GAD_males_ [.04] < GAD → DA_males_ [.41]), whereas both failure and acting out were not apparent in females (DA → GAD_females_ = GAD → DA_females_ [≤.08]) (see Table 4, middle panel). To test for this gender difference, we ran a set of Bayesian models. The structure of these models was similar to those of the Bayesian models on age group differences in failure and acting out. The models showed that the “acting out in males” model, was by far the best-fitting model (Table 5, third panel). These findings suggested acting out to be stronger in males than in females (see Table 4, middle panel: GAD → DA_males_ [.41] > GAD → DA_females_ [.03]). A follow-up set of Bayesian models confirmed this to be the case (Table 5, fourth panel).

#### Friendship Group Differences

The final three-covariate LTA model revealed friendships differences in failure and acting out across 4-year intervals: Failure was weaker than acting out in poorer-quality friendships (pqf) (DA → GAD_pqf_ [.11] < GAD → DA_pqf_ [.25]), whereas both failure and acting out were not apparent in higher-quality friendships (hqf) (DA → GAD_hqf_ = GAD → DA_hqf_ [≤.06]) (see Table 4, lower panel). To test for this friendship difference, we ran a set of Bayesian models. The structure of these models was similar to those of the Bayesian models on age group differences in failure and acting out. The models showed that the “acting out in poorer friendships” model was by far the best-fitting model (Table 5, fifth panel). These findings suggested acting out to be stronger in poorer-quality friendships (see Table 4, lower panel) (GAD → DA_pqf_ = .25) than higher-quality friendships (GAD → DA_hqf_ = .06). A follow-up set of Bayesian models confirmed this to be the case (Table 5, sixth panel).

## Discussion

The present longitudinal research set out to examine the development of co-occurring direct aggression and generalized anxiety disorder during adolescence. To what extent are adolescents aggressive, anxious, or show a combination of both high aggression and anxiety? We studied whether these co-occurrence patterns remain stable or change during adolescence. Do anxious adolescents “act out” and become aggressive over time, as the acting out model would suggest (Carlson and Cantwell 1980)? Do aggressive adolescents become more anxious over time, as the failure model (Capaldi 1992; Patterson and Stoolmiller 1992) would predict? Or is change fairly rare, as the stability model suggests? We identified four types of co-occurrence of direct aggression and anxiety in adolescence: an anxious type, an aggressive type, a comorbid aggressive type and a no problems type, and studied increases and decreases of these types, as well as patterns of intra-individual change.

Our person-centered approach allowed us to study how concrete individuals develop during adolescence. We observed a clear developmental trend. Individuals tended to grow faster out of the aggressive and comorbid aggressive types in middle-to-late adolescence than in early-to-middle adolescence. On the other hand, chances to grow into the anxious type were similar in both cohorts. These findings supported our hypothesis of “accelerated development in the older cohort”. We found one other group-based developmental pattern, namely “gender differentiation”. Gender differences in problem behaviors became stronger over time.

Support was found for two perspectives of intra-individual change in aggression/anxiety types. We observed considerable stability of the no problems type and, to a lesser extent, of the anxious type. Acting out was found in early-to-middle adolescents, males, and adolescents with poorer friendships. Acting out partly explained accelerated development and gender differentiation.

### Development of Aggression and Anxiety in Adolescence: Group-Dependent Increases and Decreases of Aggression/Anxiety Types

#### Accelerated Differential Development

The aggressive type peaked between ages 13 and 16 and decreased rapidly between ages 16 and 20. Similarly, the comorbid aggressive type decreased faster in middle-to-late adolescence than in early-to-middle adolescence. On the other hand, the anxious type showed a small but systematic increase in both cohorts. These findings were in support of our hypothesis of “accelerated development in the older cohort”. The pattern for direct aggression shows similarity to the age-crime curve of delinquency (Farrington 1986). Namely, delinquency also tends to peak in early and middle adolescence and decrease quickly thereafter. A second similarity in the developmental patterns of direct aggression and delinquency is that both show adolescence-limited and life-course persistent “offenders” (Moffitt 1993). Our findings showed that, at most, 11.5 % (prevalence of aggressive type at age 20, see Table 1) of the adolescents qualified as life-course persistent aggressive. In contrast, apart from the persistent group, 28.4 % belonged to the aggressive type during at least one wave and qualified as adolescence-limited aggressive. Obviously, most individuals grow out of direct aggression and learn alternative ways to solve problems in the second half of adolescence. This is most likely due to the fact that adolescents learn to settle conflicts without using direct aggression. For instance, their cognitive empathy increases (Van der Graaff et al. 2014) and they learn to use problem solving skills in conflicts (Van Doorn et al. 2011). The peak in vulnerability for direct aggression clearly lies in early-to-middle adolescence.

In contradistinction, GAD increased systematically during the whole of adolescence. At age 12, 11.6 % belonged to the anxious type, and this percentage rose to 21 at age 20. This finding is consistent with theorizing that assumes that various anxiety symptoms express themselves at different time periods, and that adolescence is the key period for the expression of GAD (Westenberg et al. 2001; Weems 2008). As identity and autonomy grow in adolescence (Meeus 2011), individuals come to understand their own responsibility for shaping their futures. For a substantial number of adolescents, this responsibility clearly goes together with worries about the future, a key aspect of GAD. Finally, we observed a remarkable alternation of the prevalence of the aggressive and anxious types in adolescence (Table 1): from 27.6 and 11.6 at age 12, to 11.5 and 21 % at age 20, respectively. Obviously, the importance of generalized anxiety outgrows that of aggression during adolescence. The findings also demonstrate that the persistence of the developmental risk profile for generalized anxiety is bigger than that of the developmental risk profile for aggression.

In general, our findings show that individuals are especially likely to grow out of the unadjusted aggressive and comorbid aggressive types and into the adjusted no problems type during the second half of adolescence. The increase of the no problems type is consistent with the general finding that adolescents mature during the second decade of life, as evidenced by a systematic growth of identity achievement (Meeus et al. 2010), resilient personality (Meeus et al. 2011), and personality organization (Klimstra et al. 2009), as well as upon systematic empirical evidence that identity achievement (Crocetti et al. 2009) and a resilient and well organized personality accompany less aggression and anxiety (Akse et al. 2004).

#### Gender Differentiation

In support of H2, we found gender differences in the developmental heterogeneity of aggression, comorbid aggression and generalized anxiety. The prevalence of the aggressive and comorbid aggressive types was higher over time for males, whereas that of the anxious type was higher for females. We also found that this gender difference became stronger during the five waves of our study. Our findings extend the previously noted emergence of gender differences in depression during adolescence (Nolen-Hoeksema and Girgus 1994).

### Intra-Individual Change of Aggression/Anxiety Types Over Time: Stability and Acting Out

#### Stability

We did not find general support for the stability perspective. However, we did observe considerable stability for the no problems type, in particular. First, stability of the no problems type was stronger than for the other aggression/anxiety types, and it became even stronger in middle-to-late adolescence as compared to early-to-middle adolescence. Stability of the anxious type was also substantial. Thus, the transition patterns make clear that the no problems and, to a lesser extent, the anxious type serve as normative endpoints in the development of direct aggression and generalized anxiety. Second, stabilities of the aggressive and comorbid aggressive types were substantially lower than that of the no problems type. The stability of the comorbid aggressive type was particularly low; at the end of late adolescence, only 1.6 % of the middle-to-late adolescent cohort belonged to that type. This seems to indicate a decrease in strongly co-occurring problem behavior during adolescence. At the end of their teens, the vast majority of those adolescents belonged to the pure direct aggressive or anxious type.

#### Acting Out: A Mechanism Explaining Heterogeneity in Development

We found substantial but group-dependent support for the acting out perspective. Acting out was found in early-to-middle adolescents, males, and adolescents with poorer-quality friendships. In all three groups, there were substantial transitions from the anxious type to the aggressive type during 4 years (between 20 and 41 %). Remarkably, acting out was most prevalent in subgroups that, generally speaking, are more vulnerable for aggressive behavior, namely early-to-middle adolescents and males.

Acting out can be seen as a mechanism that contributes to the explanation of the two observed processes of developmental heterogeneity in aggression and generalized anxiety: accelerated development in middle-to-late adolescence and gender differentiation. Accelerated differential development was visible in accelerated decrease of aggression in middle-to-late adolescence and not in early-to-middle adolescence. Acting out indexes growing from the anxious type into the aggressive type, and therefore partly prevents a decrease of the aggressive type in early-to-middle adolescence. This prevention effect of acting out is no longer active in middle-to-late adolescence. Thus, acting out partly explains an accelerated decrease of the aggressive type in middle-to-late adolescence. Additionally, acting out was a building block of gender differentiation. Acting out was present in males and not in females. Acting out implies growing into the aggressive type, and therefore partly explains the increase of the aggressive type in males and not in females. Thus, acting out partly explains gender differences in aggression that grow during adolescence.

The percentages of acting out (that is, of growing into the aggressive type) in early-to-middle adolescents, males, and adolescents with poorer friendships were rather substantial, and ranged between 20 and 41 %, respectively (see Table 4). For instance, in males and adolescents with poorer friendships, the percentages of acting out (.41 and .25, respectively) were larger than those of the strongest transition to grow out of aggression, that is, the transition from the aggressive into the no problems type (.30 and .19, respectively). Only in early-to-middle adolescence was the transition from the aggressive to the no problems type larger (.34 and .20, respectively). But also here, the percentage of acting out was not small.

We tend to interpret acting out as the attempt of adolescents to switch from anxiety to instrumental aggression, in order to become more visible and obtain an autonomous position in the adolescent world. Earlier research showed that anxious children and adolescents were less liked and more often rejected by peers (Baker et al. 2014; Cunningham and Ollendick 2010). The transition from the anxious into the aggressive type might therefore signify an attempt to leave this disadvantaged position. In other words, they might switch from a flight strategy into a fight strategy in order to obtain a somewhat more comfortable stance in adolescence (Kunimatsu and Marsee 2012). A related explanation has been offered by Granic (2014), who suggested that anxiety may lead to aggression through loss of inhibitory control or ego-depletion. Both explanations stress that anxiety leads to defensive aggression. Our findings showed that early-to-middle adolescents, males, and adolescents with poorer-quality friendships had greater chances to make this switch. Our interpretation is supported by findings showing, for instance, that early adolescents use visible conflict engagement strategies more often than late adolescents (Van Doorn et al. 2011), that males have negative interactions with friends more often than females (De Goede et al. 2009) and that aggression in more prevalent in poorer friendships (Card et al. 2008).

#### Absence of Failure

We did not find any support for the failure perspective. Four-year transition probabilities from the aggressive into the anxious type were 5 % in the whole sample, and lower in most of the subgroups we observed. Our findings are not consistent with several other person-centered studies that supported the failure model (Burke et al. 2005; Ialongo et al. 1994; Lahey et al. 2002; Roza et al. 2003). This may be due to the relatively weak designs employed by these studies, since none of them explicitly tested the failure model against competing co-occurrence models. In addition, our study showed acting out to be stronger than failure in poorer friendships. This finding is totally inconsistent with the assumption of Patterson and colleagues that failure is typical for poorer friendships.

### Limitations, Clinical Implications, and Suggestions for Further Research

Several limitations of the present study should be recognized. First, our study mainly presents descriptive findings and mechanisms of change and stability in aggression/anxiety types. Our findings call for studies that specify conditions that might predict the timing of these transitions.

A second limitation of the present study concerns our use of self-reported aggression. However, the correlation between aggression and anxiety was very low or absent in the present study (between .01 and .12 in the various waves). Therefore, the aggression/anxiety types were not the outcome of overlapping and biased self-reports.

A third limitation has to do with our quite specific conceptualization of aggression and anxiety behaviors. This calls for replication of the present study with various other measures of internalizing and externalizing problems.

A fourth limitation is that we are unable to compare our findings on stability of the types with those of the earlier studies. This is simply due to the fact that the earlier studies did not present percentages of stability in these co-occurrence types.

Clinical implications of the present study are twofold. First, generalized anxiety seems to be a more persistent risk factor for adolescents’ future than aggression. We found intra-individual stability of the anxious type to already be substantial in early-to-middle adolescence (.58 across 4 years). Both findings underscore the need and possibility for early identification of generalized anxiety. This is especially because we also know that early intervention for generalized anxiety can be successful and more cost-effective than clinical treatment (Dadds et al. 1997). Second, our study suggests a need to explicitly focus on both negativity and power differences in best friendships in cognitive-behavioral therapy (CBT) programs aiming to treat aggression (Lochman 1992). The capacity to develop positive and symmetrical personal relationships with peers is a key developmental task for adolescents, because these types of relationships are normative in adulthood (Youniss and Smollar 1985). Since aggressive adolescents tend to have best friendships that are also high in negativity and high in power imbalance, they are at risk for not adequately learning interpersonal problem solving skills. Skills training in handling power and negativity issues may therefore be a valuable addition to sessions of CBT aggression programs that focus on problem solving in personal relationships.

## Conclusion

The present study has contributed significantly to our understanding of change and stability of aggression/anxiety types in adolescence. It is the first study to show that types of aggression and comorbid aggression decrease during middle-to-late adolescence (accelerated development), whereas the anxious type increases regularly during the entire adolescent period. We found substantial support for the acting out perspective. We interpreted acting out as the attempt of adolescents to switch from anxiety to instrumental aggression (Kunimatsu and Marsee 2012; Granic 2014), in order to become more visible and obtain an autonomous position in the adolescent world. This tendency proved to be relatively strong in early-to-middle adolescents, males, and adolescents with poorer friendships. Acting out contributed to the explanation of accelerated development and gender differentiation in problem behavior. We also observed an increase of adolescents with no problems. This increase is consistent with the general finding that adolescents mature during the second decade of life, as has been shown, for instance, in studies into identity formation and personality development.
